# Does Inclusion of Education and Marital Status Improve SCORE Performance in Central and Eastern Europe and Former Soviet Union? Findings from MONICA and HAPIEE Cohorts

**DOI:** 10.1371/journal.pone.0094344

**Published:** 2014-04-08

**Authors:** Olga Vikhireva, Grazyna Broda, Ruzena Kubinova, Sofia Malyutina, Andrzej Pająk, Abdonas Tamosiunas, Zdena Skodova, Galina Simonova, Martin Bobak, Hynek Pikhart

**Affiliations:** 1 Epidemiology and Public Health Department, University College London, London, United Kingdom; 2 Department of CVD Epidemiology, Prevention, and Health Promotion, the Cardinal Stefan Wyszynski Institute of Cardiology, Warsaw, Poland; 3 Environmental Health Monitoring System, National Institute of Public Health, Prague, Czech Republic; 4 Institute of Internal and Preventive Medicine, Siberian Branch of the Russian Academy of Medical Sciences, Novosibirsk, Russia; 5 State Novosibirsk Medical University, Novosibirsk, Russia; 6 Department of Epidemiology and Population Sciences, Institute of Public Health, Faculty of Health Sciences, Jagiellonian University Medical College, Krakow, Poland; 7 Laboratory of Population Research, Institute of Cardiology, Lithuanian University of Health Sciences, Kaunas, Lithuania; 8 Preventive Cardiology Department, Institute of Clinical and Experimental Medicine, Prague, Czech Republic; Bielefeld Evangelical Hospital, Germany

## Abstract

**Background and Objective:**

The SCORE scale predicts the 10-year risk of fatal atherosclerotic cardiovascular disease (CVD), based on conventional risk factors. The high-risk version of SCORE is recommended for Central and Eastern Europe and former Soviet Union (CEE/FSU), due to high CVD mortality rates in these countries. Given the pronounced social gradient in cardiovascular mortality in the region, it is important to consider social factors in the CVD risk prediction. We investigated whether adding education and marital status to SCORE benefits its prognostic performance in two sets of population-based CEE/FSU cohorts.

**Methods:**

The WHO MONICA (MONItoring of trends and determinants in CArdiovascular disease) cohorts from the Czech Republic, Poland (Warsaw and Tarnobrzeg), Lithuania (Kaunas), and Russia (Novosibirsk) were followed from the mid-1980s (577 atherosclerotic CVD deaths among 14,969 participants with non-missing data). The HAPIEE (Health, Alcohol, and Psychosocial factors In Eastern Europe) study follows Czech, Polish (Krakow), and Russian (Novosibirsk) cohorts from 2002–05 (395 atherosclerotic CVD deaths in 19,900 individuals with non-missing data).

**Results:**

In MONICA and HAPIEE, the high-risk SCORE ≥5% at baseline strongly and significantly predicted fatal CVD both before and after adjustment for education and marital status. After controlling for SCORE, lower education and non-married status were significantly associated with CVD mortality in some samples. SCORE extension by these additional risk factors only slightly improved indices of calibration and discrimination (integrated discrimination improvement <5% in men and ≤1% in women).

**Conclusion:**

Extending SCORE by education and marital status failed to substantially improve its prognostic performance in population-based CEE/FSU cohorts.

## Introduction

The SCORE (Systematic COronary Risk Evaluation) scale is a widely used instrument for predicting the risk of future cardiovascular disease (CVD) across European populations [1]. It estimates the 10-year risk of cardiovascular mortality based on age, gender, blood lipids, blood pressure (BP), and smoking, in 40–65-year-olds free of manifested CVD. Two versions of SCORE were created for high- and low-risk European countries. The European Society of Cardiology (ESC) recommends applying the high-risk SCORE to the populations of Central and Eastern Europe (CEE) and former Soviet Union (FSU) [2], although this version was derived without reference to local data.

As SCORE includes only conventional cardiovascular risk factors, there have been ongoing attempts to improve its performance by adding resting heart rate [3], high-density lipoprotein cholesterol [4], and other factors [5], [6] to the model. Across CEE/FSU populations, the majority of which still face high levels of fatal CVD [7], pronounced and increasing socioeconomic and sociodemographic differentials in all-cause and cardiovascular mortality have been reported [8]–[10]. Moreover, education and marital status have been shown to independently predict cardiovascular risk in CEE/FSU populations [11]–[13]. These two easily and routinely assessed parameters reflect different pathways between social circumstances and CVD. Education can act via life-style behaviours, problem-solving abilities, and acquisition of positive social, psychological and economic skills and assets [14], [15], while marital status can affect CVD risk through social connections, a sense of social and familial role, socioeconomic support, and facilitation of health-promoting behaviours [16]–[19]. Therefore, education and marital status are likely candidates for inclusion in cardiovascular risk models, together with conventional risk factors. However, to the best of our knowledge, the prognostic performance of CVD risk scales extended by these characteristics, or other socioeconomic and sociodemographic parameters, has not been assessed in CEE/FSU.

The aim of our study was to investigate whether SCORE calibration and discrimination improve after extension by such social indicators as education and marital status, using two sets of population-based CEE/FSU cohorts.

## Methods

### Study Population and Samples

We used the data from two international multi-centre studies – the World Health Organization (WHO) MONICA (MONItoring of trends and determinants in CArdiovascular disease) Project [20], [21] and HAPIEE (Health, Alcohol, and Psychosocial factors In Eastern Europe) study [22].

The WHO MONICA Project monitored the trends in CVD rates and risk factors in 38 populations from the mid-1980s to at least the mid-1990s [20], [21]. Risk factors were assessed in random population samples of men and women aged 35–64. In some centres, the study samples were followed for mortality. We obtained the baseline data and the data on subsequent 10-year mortality for the following MONICA samples: the Czech sample from six country districts examined in 1992; Polish Warsaw and Tarnobrzeg samples screened in 1983–84 and 1987–89; Lithuanian Kaunas samples examined in 1983–85, 1986–87, and 1992–93; and Russian Novosibirsk samples screened in 1985–86, 1988–89, and 1994–95. The numbers of subjects and response rates are shown in **Table 1**. At baseline, participants completed a questionnaire survey, underwent a clinical examination, and provided a blood sample. The mortality follow-up used the data from national and local mortality registers [20], [21].

**Table 1:** Description of participating cohorts and selection of analytical samples.

	*MONICA*					*HAPIEE*		
	CZ	PL-W	PL-T	LT	RU	CZ	PL-K	RU
Baseline, year (response rate, %)	1992 (65)	1983–84 (74),1988–89 (70)	1983–84 (82),1987–88 (73)	1983–85 (70),1986–87 (70),1992–93 (59)	1985–86 (72),1988–89 (73),1994–95 (72)	2002–05 (55)	2002–05 (61)	2002–05 (61)
Recruited, N	3,273	4,079	4,033	5,968	9,835	8,856	10,728	9,363
Within the study age range, N	1,977	3,396	3,274	4,596	6,062	8,781	10,728	9,352
No pre-existing CVD, N	1,861	2,437	2,782	3,806	5,669	7,633	8,316	7,316
Non-missing SCORE values, N	1,340	2,404	2,729	3,301	5,253	6,018	7,209	7,290
Non-missing values of SCORE,education, and marital status, N	1,337	2,404	2,729	3,291	5,208	5,850	6,812	7,238

CZ – Czech Republic; LT – Lithuania; PL-K – Poland (Krakow); PL-T – Poland (Tarnobrzeg); PL-W – Poland (Warsaw); RU – Russia.

HAPIEE is a multi-centre study of CVD and other chronic conditions in CEE/FSU [22]. It follows random population samples of men and women aged 45–69 at baseline (2002–05) from the Czech Republic (Havířov/Karviná, Hradec Králové, Jihlava, Kroměříž, Liberec, and Ústí nad Labem), Poland (Krakow), Russia (Novosibirsk), and Lithuania (Kaunas). The four cohorts have been followed for cause-specific mortality and non-fatal CVD. As the Lithuanian cohort was established several years later and had fewer CVD deaths, it was not included in our analyses. The baseline data collection included a structured questionnaire survey and physical examination, with a fasting venous blood sample collection. In Czech and Polish HAPIEE, the study questionnaire was completed at home, prior to medical examination in a clinic. This explains the smaller proportion of Czech and Polish participants with non-missing data. Mortality data come from national (the Czech Republic) and local (Poland and Russia) registers [22].

As SCORE predicts cardiovascular risk in individuals over 40 and without pre-existing atherosclerotic CVD [1], we excluded subjects aged <40 at baseline and those with medical evidence or a self-reported history of doctor-diagnosed myocardial infarction, angina, or stroke. Overall, data on conventional, SCORE-comprising risk factors, education, and marital status were available for 14,969 MONICA subjects and 19,900 HAPIEE participants (**Table 1**).

### Ethics Statement

The MONICA protocol was approved by the local ethics committees in each participating country: the Institute of Clinical and Experimental Medicine Ethics Committee (Prague, the Czech Republic), the National Institute of Cardiology Ethics Committee (Warsaw, Poland), the Jagellonian University Ethics Committee (Krakow, Poland), the Kaunas University Ethics Committee (Kaunas, Lithuania), and the Institute of Internal and Preventive Medicine Ethics Committee (Novosibirsk, Russia) [20]. The HAPIEE protocol was approved by the University College London/University College London Hospital Ethics Committee (London, UK) and by local ethics committees at each study centre: the National Institute of Public Health Ethics Committee (Prague, the Czech Republic), the Jagellonian University Ethics Committee (Krakow, Poland), and the Institute of Internal and Preventive Medicine Ethics Committee (Novosibirsk, Russia) [22]. Both studies were conducted according to the standards of the Declaration of Helsinki. All participants provided written informed consent.

### Measurements

The SCORE risk predictors include age, sex, smoking status, total cholesterol (TC), and systolic BP (SBP). The measurement of these parameters in MONICA and HAPIEE participants is described in detail elsewhere [20], [22], [23]. Individuals currently and regularly smoking at least one cigarette per day were regarded as current smokers; never and ex-smokers were considered non-smokers, according to the SCORE criteria [1]. SBP and TCH measurement was subjected to extensive quality control.

In the original categories of education and marital status (five and four categories, respectively), the numbers of atherosclerotic CVD deaths were not sufficient for adequately powered analyses. Therefore, these factors were dichotomised and defined as “higher” (university, secondary, or vocational) vs. “lower” (primary or less) education and “married” (married/cohabiting) vs. “non-married” (single, divorced/separated, or widowed) status. In line with the SCORE end-points [1], the study outcome was atherosclerotic cardiovascular death (International Classification of Diseases (ICD) 9 codes: 401–414, 426–443 (except 426.7, 429.0, 430.0, 432.1, 437.3, 437.4, and 437.5), 798.1 and 798.2; ICD-10 codes: I10-I15, I20-I25, I44-I73 (except I45.6, I51.4, I52, I60, I62, I67.1, I67.5 and I67.7), R96.0, and R96.1).

### Statistical Analyses

The high-risk version of the SCORE scale, recommended by the ESC for CEE/FSU populations [2], was used to predict the risk of fatal atherosclerotic CVD in all MONICA and HAPIEE samples. The recently introduced Czech and Polish SCORE versions were not used, as they lack a detailed description of their development and recalibration [24], [25].

The prognostic performance of risk prediction scales, such as SCORE, could be assessed via calibration and discrimination [26], [27]. Calibration reflects the closeness between predicted and observed risks. Discrimination shows how accurately the participants who will experience events (such as fatal CVD) during the follow-up are separated from those who will remain event-free. Better calibration and discrimination are denoted, respectively, by lower χ^2^ values and higher *p*-values in the Hosmer-Lemeshow test [28] and higher values of the Harrell’s C-statistic [29]. The additional prognostic information, provided by extra risk predictors, could be assessed in likelihood ratio tests (LRT). Lower LRT *p*-values denote more pronounced differences between the nested baseline and extended models and, hence, better predictive performance of the latter. More clinically relevant parameters of risk reclassification are net reclassification index and integrated discrimination improvement (IDI). As our additional risk factors were dichotomized and, therefore, specific to MONICA and HAPIEE samples, we used IDI, which is relatively independent of risk thresholds and categories and reflects the extended model’s ability to improve average sensitivity without compromising average specificity [30], [31].

We analysed the prognostic ability of the extended SCORE separately for men and women in each MONICA and HAPIEE sample. First, we explored the role of SCORE, education, and marital status as fatal CVD predictors, using Cox regression models. We then investigated calibration and discrimination of the SCORE extended by education and marital status, calculating Hosmer-Lemeshow χ^2^, Harrell’s C, LRT *p*, and IDI. The SCORE performance was compared for the baseline Model 1 (SCORE only) vs. Model 2 (SCORE and education), Model 3 (SCORE and marital status), and Model 4 (SCORE, education, and marital status).

The use of Cox proportional hazards regression models was justified by the high *p*-values in Schoenfeld’s test. The data from all MONICA waves were pooled within samples, as the SCORE-fatal CVD association demonstrated no evidence of confounding by or statistical interaction with the study wave. The competing-risk regression analyses [32], accounting for the risk of death from causes other than atherosclerotic CVD, produced very similar results (not presented) to those of the standard Cox analyses. No significant interactions between SCORE and additional risk factors were detected. Simultaneously extending SCORE by education and marital status was possible, due to low values of phi correlation coefficient (not presented). To enable comparisons between non-extended and extended models, the analyses included only subjects with known values of conventional and additional risk factors. Additional sensitivity analyses used multiply imputed data for the samples with the highest proportion of missing baseline SCORE values – Czech MONICA, Lithuanian MONICA, Czech HAPIEE, and Polish HAPIEE. The imputation model, which was based on the chained equations approach [33] and generated 10 imputations, included SCORE, education, marital status, atherosclerotic CVD death, and logarithm of survival time.

The SCORE calibration in HAPIEE was affected by the fact that the current follow-up of HAPIEE samples is less than 10 years (**Table 2**). However, we focused on the changes in calibration after the SCORE extension by education and marital status, rather than on SCORE calibration *per se*. As the Hosmer-Lemeshow test quantifies the agreement between predicted and observed events across risk deciles, it was applied to the non-dichotomised SCORE, which treats individual levels of absolute risk (percentages in the respective SCORE chart cells) as a continuous variable. All statistical analyses were performed using Stata/IC 12.0 (StataCorp LP, Texas, USA).

**Table 2:** Descriptive characteristics of the study samples.

	*MONICA men*					*HAPIEE men*		
	CZ	PL-W	PL-T	LT	RU	CZ	PL-K	RU
N	635	1,253	1,267	1,648	2,550	2,590	3,262	3,214
Mean age (SD), years	50.7 (7.1)	51.9 (7.0)	52.5 (6.8)	51.3 (7.1)	52.3 (6.8)	57.9 (7.1)	57.1 (6.9)	57.5 (7.0)
Age groups, %								
40–44	27.1	21.6	17.4	23.7	17.7			
45–49	22.5	20.8	20.8	19.4	22.6	17.2	20.6	18.9
50–54	20.9	20.0	21.6	18.1	20.8	19.8	21.4	21.3
55–59	15.9	22.5	23.2	22.9	23.1	20.9	21.8	21.6
60–64	13.5	15.1	17.0	15.9	15.8	21.5	18.5	18.2
65+						20.7	17.8	20.0
Current smoking, %	40.2	52.5	56.3	35.1	53.1	26.7	32.8	51.2
Mean TC (SD), mmol/l	6.2 (1.3)	5.6 (1.0)	5.5 (1.0)	6.0 (1.2)	5.5 (1.2)	5.7 (1.0)	5.8 (1.1)	6.0 (1.2)
Mean SBP (SD), mm Hg	139.0 (20.8)	142.2 (23.8)	136.6 (21.2)	137.3 (20.1)	136.3 (20.2)	143.8 (18.5)	141.6 (20.2)	141.6 (22.6)
Lower education ^a^, %	63.5	46.3	71.0	37.7	43.3	5.1	8.1	10.6
Non-married status ^b^, %	15.1	11.5	8.4	7.3	8.4	15.3	12.1	12.0
Median follow-up (IQR), years	10 (10–10)	10 (10–10)	10 (10–10)	10 (10–10)	10 (10–10)	8.1 (7.7–8.9)	7.1 (6.9–7.7)	6.2 (5.7–7.0)
Atherosclerotic CVD deaths, N (%)	31 (4.9)	86 (6.9)	62 (4.9)	58 (3.5)	178 (7.0)	62 (2.4)	55 (1.7)	160 (5.0)
	***MONICA women***					***HAPIEE women***		
	**CZ**	**PL-W**	**PL-T**	**LT**	**RU**	**CZ**	**PL-K**	**RU**
N	702	1,151	1,462	1,643	2,658	3,260	3,550	4,024
Mean age (SD), years	51.2 (7.2)	51.8 (7.0)	52.4 (6.9)	50.9 (7.1)	52.2 (7.0)	57.4 (7.0)	56.5 (6.9)	57.3 (7.1)
Age groups, %								
40–44	24.1	21.8	18.3	24.7	19.4			
45–49	24.9	21.6	21.6	20.9	22.0	19.6	22.7	20.7
50–54	17.5	20.4	21.4	19.7	20.8	21.1	23.6	20.8
55–59	18.1	20.9	21.1	19.4	20.5	18.3	20.8	21.8
60–64	15.4	15.3	17.6	15.4	17.3	23.7	17.6	17.1
65+						17.3	15.3	19.7
Current smoking, %	20.7	31.2	7.5	4.2	3.4	21.2	25.6	10.1
Mean TC (SD), mmol/l	6.3 (1.3)	5.7 (1.0)	5.7 (1.1)	6.3 (1.2)	5.9 (1.3)	5.9 (1.0)	5.9 (1.1)	6.5 (1.3)
Mean SBP (SD), mm Hg	137.6 (21.8)	142.6 (25.1)	141.6 (24.3)	137.2 (22.7)	138.5 (22.6)	134.1 (19.3)	133.2 (20.9)	141.6 (25.7)
Lower education ^a^, %	64.4	41.7	79.8	38.0	36.1	16.9	11.5	8.9
Non-married status ^b^, %	22.4	28.0	16.8	17.7	27.0	30.7	31.5	39.4
Median follow-up (IQR), years	10 (10–10)	10 (10–10)	10 (10–10)	10 (10–10)	10 (10–10)	8.2 (7.8–8.9)	7.1 (6.9–7.8)	6.7 (6.0–7.1)
Atherosclerotic CVD deaths, N (%)	13 (1.9)	21 (1.8)	19 (1.3)	25 (1.5)	84 (3.2)	27 (0.8)	34 (1.0)	57 (1.4)

CZ – Czech Republic; LT – Lithuania; PL-K – Poland (Krakow); PL-T – Poland (Tarnobrzeg); PL-W – Poland (Warsaw); RU – Russia. IQR – interquartile range; N/A – not applicable; SBP – systolic blood pressure; SD – standard deviation; TC – total cholesterol. ^a^ Primary or incomplete primary education. ^b^ Single, divorced/separated, and widowed status.

## Results

### Description of the Study Samples

The baseline characteristics of our analytical samples are presented in **Table 2**. The mean age of participants was close to 52 years in MONICA and 57 years in HAPIEE, with relatively similar sizes of the five-year age groups. Smoking prevalence was high in both studies, with the exception of MONICA women from Tarnobrzeg, Kaunas, and Novosibirsk. Czech and Polish men participating in HAPIEE smoked less than their MONICA counterparts. By contrast, among Russian women, smoking prevalence was higher in HAPIEE than in MONICA. In both studies, mean levels of TC were close to 6 mmol/l and tended to be slightly higher in women. The highest mean SBP levels, exceeding 140 mm Hg, were observed for MONICA participants from Warsaw and Czech HAPIEE men. The proportion of lower-educated people was markedly higher in MONICA than in HAPIEE. In both studies, the majority of participants were married. The median follow-up duration was 10 years in MONICA and 6.2–8.2 years in HAPIEE (**Table 2**). The highest percentage of atherosclerotic CVD deaths was observed for Russian MONICA and HAPIEE men. In women, the observed risk of fatal CVD was lower, but reflected the same ranking.

### SCORE, Education, and Marital Status as Predictors of Fatal Atherosclerotic CVD

As shown in **Tables 3**–**4**, in most MONICA and HAPIEE samples, the high-risk SCORE ≥5% at baseline was a significant predictor of atherosclerotic CVD mortality, both before and after controlling for education, marital status, or both (adjusted hazard ratios (HR) 1.5–5.8 for MONICA and 2.7–8.5 for HAPIEE). After adjustment for SCORE, a significant association between fatal CVD and education was demonstrated in four out of eight male MONICA and HAPIEE samples; for the association between fatal CVD and marital status, this figure was five out of eight (**Table 3**). For female MONICA and HAPIEE samples, the respective figures were three out of eight and one out of eight (**Table 4**).

**Table 3:** Prognostic performance of the high-risk SCORE (≥5% vs. <5%) in MONICA and HAPIEE men before and after inclusion of education (lower vs. higher) and marital status (non-married vs. married).

	*MONICA men*					*HAPIEE men*		
	CZ	PL-W	PL-T	LT	RU	CZ	PL-K	RU
**Model 1** (SCORE only)								
SCORE HR	5.32	4.50	3.06	3.99	2.66	8.86	3.48	7.05
(95% CI)	(2.30–12.30)	(2.68–7.56)	(1.77–5.29)	(2.24–7.10)	(1.96–3.62)	(3.22–24.42)	(1.70–7.10)	(3.72–13.37)
Hosmer-Lemeshow χ^2^ (*p*) ^a^	17.15 (0.02)	13.89 (0.05)	4.62 (0.71)	20.55 (<0.01)	4.41 (0.73)	11.93 (0.15)	5.91 (0.55)	46.92 (<0.01)
Harrell’s C	0.69	0.67	0.63	0.66	0.62	0.66	0.63	0.63
Model 2 (SCORE and education)								
SCORE HR	5.35	4.32	2.90	3.75	2.43	8.35	3.34	6.62
(95% CI)	(2.31–12.38)	(2.56–7.27)	(1.67–5.05)	(2.08–6.76)	(1.78–3.33)	(3.03–23.03)	(1.63–6.84)	(3.48–12.58)
Education HR	1.20	1.56	1.44	1.30	1.70	3.33	1.98	1.68
(95% CI)	(0.58–2.49)	(1.02–2.41)	(0.76–2.73)	(0.77–2.20)	(1.25–2.30)	(1.69–5.56)	(0.97–4.06)	(1.13–2.49)
Hosmer-Lemeshow χ^2^ (*p*) ^a^	9.38 (0.23)	10.69 (0.22)	9.16 (0.33)	18.01 (0.02)	4.52 (0.81)	13.83 (0.09)	12.35 (0.14)	25.99 (<0.01)
Harrell’s C	0.70	0.69	0.65	0.69	0.65	0.70	0.64	0.64
LRT *p*	0.72	0.04	0.24	0.33	<0.01	<0.01	<0.01	0.02
IDI, % (*p*)	–0.01 (0.78)	0.33 (0.13)	0.13 (0.17)	<0.01 (0.99)	0.36 (0.01)	0.66 (0.03)	0.13 (0.16)	0.24 (0.04)
Model 3 (SCORE and marital status)								
SCORE HR	5.73	4.55	3.05	4.05	2.70	9.09	3.43	7.07
(95% CI)	(2.46–13.33)	(2.71–7.65)	(1.76–5.28)	(2.28–7.21)	(1.99–3.68)	(3.30–25.04)	(1.68–7.01)	(3.73–13.41)
Marital status HR	5.13	2.01	1.17	1.93	1.73	1.83	1.84	2.09
(95% CI)	(2.51–10.50)	(1.17–3.47)	(0.50–2.71)	(0.88–4.26)	(1.12–2.68)	(1.01–3.33)	(0.95–3.55)	(1.43–3.07)
Hosmer–Lemeshow χ^2^ (*p*) ^a^	13.84 (0.05)	18.75 (0.02)	6.74 (0.46)	28.40 (<0.01)	5.74 (0.57)	6.51 (0.59)	9.94 (0.27)	35.13 (<0.01)
Harrell’s C	0.76	0.69	0.64	0.68	0.64	0.69	0.65	0.65
LRT *p*	<0.01	0.02	0.73	0.13	0.02	0.06	0.06	<0.01
IDI, % (*p*)	4.11 (<0.01)	0.37 (0.20)	–0.01 (0.91)	0.19 (0.26)	0.20 (0.12)	0.18 (0.14)	0.11 (0.16)	0.44 (<0.01)
**Model 4** (SCORE, education, and marital status)								
SCORE HR	5.77	4.37	2.90	3.82	2.48	8.50	3.31	6.67
(95% CI)	(2.47–13.43)	(2.59–7.36)	(1.67–5.05)	(2.12–6.88)	(1.81–3.40)	(3.08–23.46)	(1.62–6.78)	(3.51–12.68)
Education HR	1.16	1.59	1.44	1.29	1.66	3.07	1.95	1.63
(95% CI)	(0.56–2.42)	(1.03–2.44)	(0.76–2.72)	(0.76–2.18)	(1.23–2.25)	(1.54–6.10)	(0.95–3.99)	(1.10–2.42)
Marital status HR	5.14	2.05	1.14	1.92	1.76	1.64	1.81	2.05
(95% CI)	(2.51–10.52)	(1.19–3.53)	(0.49–2.65	(0.87–4.23)	(1.13–2.72)	(0.90–3.00)	(0.93–3.50)	(1.40–3.01)
Hosmer-Lemeshow χ^2^ (*p*) ^a^	17.86 (0.01)	4.00 (0.86)	11.79 (0.16)	20.62 (0.01)	6.47 (0.59)	9.38 (0.31)	9.91 (0.27)	30.60 (<0.01)
Harrell’s C	0.74	0.71	0.65	0.70	0.66	0.72	0.67	0.67
LRT *p*	<0.01	0.01	0.48	0.21	<0.01	<0.01	<0.01	<0.01
IDI, % (*p*)	4.07 (0.01)	0.74 (0.06)	0.13 (0.21)	0.17 (0.31)	0.55 (0.01)	0.80 (0.02)	0.22 (0.05)	0.69 (<0.01)

CZ – Czech Republic; LT – Lithuania; PL-K – Poland (Krakow); PL-T – Poland (Tarnobrzeg); PL-W – Poland (Warsaw); RU – Russia. IDI – integrated discrimination improvement; LRT – likelihood ratio test; N/A – not applicable. ^a^ Calculated for continuous high-risk SCORE.

**Table 4:** Prognostic performance of the high-risk SCORE (≥5% vs. <5%) in MONICA and HAPIEE women before and after inclusion of education (lower vs. higher) and marital status (non-married vs. married).

	*MONICA women*					*HAPIEE women*		
	CZ	PL-W	PL-T	LT	RU	CZ	PL-K	RU
**Model 1** (SCORE only)								
SCORE HR	5.02	1.70	4.15	5.07	6.32	2.94	3.80	6.21
(95% CI)	(1.64–15.36)	(0.57–5.06)	(1.49–11.52)	(2.20–11.66)	(4.08–9.79)	(1.35–6.42)	(1.94–7.60)	(3.58–10.76)
Hosmer–Lemeshow χ^2^ (*p*) a	7.89 (0.10)	8.42 (0.08)	11.19 (0.03)	19.17 (<0.01)	14.92 (<0.01)	7.90 (0.16)	6.26 (0.18)	4.90 (0.43)
Harrell’s C	0.64	0.54	0.59	0.62	0.65	0.59	0.64	0.64
**Model 2** (SCORE and education)								
SCORE HR	4.72	1.62	4.26	3.78	5.44	2.81	3.49	5.63
(95% CI)	(1.53–14.58)	(0.54–4.80)	(1.52–11.96)	(1.60–8.90)	(3.44–8.60)	(1.28–6.17)	(1.74–7.02)	(3.20–9.89)
Education HR	1.55	4.48	0.82	4.35	1.70	1.54	1.89	1.88
(95% CI)	(0.42–5.69)	(1.64–12.23)	(0.27–2.49)	(1.70–11.09)	(1.09–2.66)	(0.65–3.67)	(0.84–4.26)	(0.99–3.55)
Hosmer–Lemeshow χ^2^ (*p*) a	5.80 (0.45)	7.09 (0.31)	13.93 (0.03)	9.10 (0.11)	11.75 (0.04)	1.74 (0.88)	2.01 (0.73)	7.02 (0.22)
Harrell’s C	0.70	0.69	0.60	0.74	0.70	0.62	0.67	0.67
LRT *p*	0.50	<0.01	0.73	<0.01	0.03	0.35	0.35	0.06
IDI, % *(p)*	–0.08 (0.73)	0.91 (0.17)	0.02 (0.61)	0.70 (0.03)	0.17 (0.36)	0.03 (0.53)	0.08 (0.38)	0.16 (0.22)
**Model 3** (SCORE and marital status)								
SCORE HR	4.96	1.61	4.30	4.98	6.22	2.85	3.82	5.81
(95% CI)	(1.62–15.17)	(0.54–4.79)	(1.53–12.08)	(2.16–11.49)	(3.98–9.71)	(1.30–6.22)	(1.92–7.61)	(3.34–10.10)
Marital status HR	2.15	1.90	0.77	1.26	1.22	1.72	1.04	1.94
(95% CI)	(0.70–6.58)	(0.80–4.52)	(0.22–2.68)	(0.50–3.15)	(0.77–1.91)	(0.81–3.68)	(0.51–2.12)	(1.14–3.30)
Hosmer–Lemeshow χ^2^ (*p*) a	12.07 (0.03)	15.33 (0.02)	6.54 (0.09)	12.12 (0.02)	21.25 (<0.01)	14.44 (0.03)	5.73 (0.33)	10.69 (0.15)
Harrell’s C	0.71	0.58	0.58	0.64	0.68	0.65	0.63	0.63
LRT *p*	0.19	0.16	0.67	0.89	0.37	0.17	0.17	0.01
IDI, % *(p)*	0.13 (0.75)	0.22 (0.14)	0.06 (0.09)	–0.01 (0.47)	–0.01 (0.92)	0.06 (0.38)	<0.01 (0.67)	0.38 (<0.01)
**Model 4** (SCORE, education, and marital status)								
SCORE HR	4.67	1.53	4.42	3.77	5.44	2.72	3.48	5.33
(95% CI)	(1.51–14.43)	(0.51–4.56)	(1.55–12.57)	(1.60–8.91)	(3.42–8.66)	(1.23–5.99)	(1.72–7.04)	(3.03–9.38)
Education HR	1.54	4.50	0.81	4.35	1.64	1.51	1.89	1.78
(95% CI)	(0.42–5.67)	(1.65–12.29)	(0.27–2.48)	(1.70–11.09)	(1.05–2.58)	(0.63–3.60)	(0.84–4.25)	(0.95–3.36)
Marital status HR	2.16	1.92	0.77	1.03	1.22	1.70	1.02	1.89
(95% CI)	(0.71–6.60)	(0.81–4.57)	(0.22–2.68)	(0.39–2.75)	(0.77–1.91)	(0.79–3.64)	(0.50–2.08)	(1.11–3.22)
Hosmer-Lemeshow χ^2^ (*p*) a	7.74 (0.26)	7.22 (0.51)	5.39 (0.37)	9.21 (0.24)	11.54 (0.12)	10.49 (0.16)	2.35 (0.89)	4.62 (0.71)
Harrell’s C	0.75	0.71	0.57	0.74	0.71	0.66	0.68	0.68
LRT *p*	0.34	<0.01	0.86	0.01	0.06	0.26	0.26	0.01
IDI, % *(p)*	0.11 (0.83)	1.17 (<0.01)	0.08 (0.18)	0.70 (0.03)	0.19 (0.38)	0.10 (0.30)	0.08 (0.38)	0.52 (0.01)

CZ – Czech Republic; LT – Lithuania; PL-K – Poland (Krakow); PL-T – Poland (Tarnobrzeg); PL-W – Poland (Warsaw); RU – Russia. IDI – integrated discrimination improvement; LRT – likelihood ratio test; N/A – not applicable.

a Calculated for continuous high-risk SCORE.

### Calibration and Discrimination of SCORE Extended by Education and Marital Status

In most MONICA samples, the extension of SCORE by education slightly reduced Hosmer-Lemeshow’s χ*^2^* values, which indicated a modest improvement in the SCORE calibration (**Tables 3**–**4**). The addition of marital status to SCORE improved calibration only in Czech men, Polish women from Tarnobrzeg, and Lithuanian women. Across most HAPIEE samples, adding education and marital status to SCORE somewhat improved its calibration.

A slight increase in Harrell’s C-statistic was observed for MONICA and HAPIEE men and women, once education and marital status were added to SCORE. According to the LRT results, the extended SCORE demonstrated improved discrimination in some MONICA and HAPIEE samples. However, in both studies, the values of IDI were <5% in men and ≤1% in women, which suggests a modest improvement in SCORE performance.

Overall, calibration and discrimination of the original, non-extended SCORE appeared to be similar, or only marginally worse, compared to the SCORE modifications extended by education and/or marital status.

## Discussion

To the best of our knowledge, this is the first attempt to assess the prognostic performance of SCORE extended by education and marital status in CEE/FSU. The high-risk SCORE significantly predicted atherosclerotic cardiovascular mortality in two sets of population-based cohorts, both before and after adjustment for additional risk factors. Education and marital status were significantly and independently from SCORE associated with fatal CVD in some samples. However, adding these factors to SCORE did not substantially improve its calibration and discrimination, which justifies the use of the original, non-extended scale.

### Strengths and Limitations

Several methodological issues should be considered when interpreting our results. First, we used the data from two separate studies, which covered different historical periods, characterized by major albeit heterogeneous changes in several domains: conventional cardiovascular risk factors (for example, smoking prevalence declined among Czech men, while it increased among Russian women [34]); socioeconomic and sociodemographic characteristics (such as improved educational attainment [7], but also rising income inequalities and decreasing proportion of married/cohabiting people [35]); and diverging trends in cardiovascular mortality after 1990 [7], which cannot be explained by the change from the ICD-9 to ICD-10 classification in the late 1990s [36]–[41]. We acknowledge that, since our data come from two sets of studies in population samples, our estimates of a longer-term mortality risk may not fully represent the trends in national rates.

Second, although MONICA and HAPIEE samples are not representative for the whole countries (for example, they are predominantly urban), they are the best available CEE/FSU sources of individual-level cohort data on the levels of CVD risk factors and mortality in the 1980–2000s. The comparability of MONICA and HAPIEE data was high, due to the similarity of the study protocols.

Third, as in most epidemiological studies, both MONICA and HAPIEE participants tended to be healthier and more affluent than non-responders. This potential discrepancy could be enhanced by the complete case analyses and dilute the association of interest. The available multiple imputation methods use the assumption of data missing (completely) at random [42], [43]. However, this assumption was unlikely to be met in our samples, as suggested by the typically higher levels of total and CVD mortality across study- and country-specific subgroups with missing vs. available SCORE. While excluding the observations with non-randomly missing values is not entirely bias-free, in our sensitivity analyses the Cox regression results across the samples with the highest SCORE missingness were similar for complete and multiply imputed data (not presented). Therefore, the possible selection bias due to non-response and SCORE missingness and the resulting potential underestimation of the strength of the association between CVD risk factors and mortality were unlikely to be substantial.

Fourth, education and marital status are only two parameters out of the wide range of social indicators which potentially influence CVD risk. For example, material deprivation may have strong effects on cardiovascular mortality [44], [45]. However, we focused on education and marital status, as they are collected in most epidemiological and clinical studies and are easily measurable, even in the primary care settings. Moreover, education and marital status reflect different pathways between social circumstances and CVD, and they have both been shown to independently predict cardiovascular risk across CEE/FSU populations [11]–[13]. The dichotomisation of education and marital status, while increasing the risk of residual confounding, was necessary in order to obtain sufficient numbers of atherosclerotic CVD deaths across exposure categories (see ***Methods***). The marked decrease in the prevalence of lower education between MONICA and HAPIEE cohorts (for example, from 64% in Czech MONICA men to 5% in Czech HAPIEE men) reflects improved access to further education in more recent birth cohorts [7], as well as the urban nature of the HAPIEE cohort (Czech MONICA sample was not exclusively urban). Some differences in the contextual meaning of lower education or non-married status across countries and over time, specifically in terms of their influence on cardiovascular risk, are possible. Nonetheless, these potential differences were unlikely to substantially affect the magnitude of the associations between socioeconomic parameters, SCORE, and fatal CVD, as our analyses were study- and country-specific.

Finally, the baseline levels of conventional and additional risk factors were likely to change during the follow-up period and, therefore, result in potential regression dilution bias, or underestimation of the association of interest [46]. However, the estimation of the future outcome risk based on the current exposure levels agrees with the general concept of risk prediction.

### Consistency with other Studies

The validity of our findings, despite the potential limitations discussed above, is supported by the fact that the levels of major risk factors and CVD mortality in MONICA and HAPIEE samples reflect the respective national cross-sectional estimates and trends presented in the WHO Global InfoBase [34] and WHO systematic reviews and reports [47]–[49].

SCORE significantly predicted cardiovascular risk, independently of social characteristics, not only across MONICA and HAPIEE samples, but also in adults from Austria [50] and Greece [51]. In most MONICA and HAPIEE samples, lower education and non-married status were linked to an increase in CVD risk even after controlling for SCORE. This agrees with the results for male participants of the Russian Lipid Research Clinics (LRC) Study [11] and MONICA-Novosibirsk [12], as well as with the findings from USA [52], [53], Finland [54], the Netherlands [55], and UK [18], [56], [57].

The lack of statistically significant, SCORE-independent associations between fatal CVD and education or marital status in most MONICA and HAPIEE samples could be partly explained by the limited numbers of atherosclerotic cardiovascular deaths. Moreover, the mechanisms of adverse effects of lower education and non-married status on cardiovascular health involve conventional risk factors, such as smoking [56]. Controlling for these factors, captured by SCORE, might over-adjust the association between education, marital status, and CVD mortality.

There is an extensive evidence of a marked and increasing social gradient in all-cause and cardiovascular mortality in CEE/FSU populations [8]–[10], [12], [58]. Therefore, we hypothesized that education and marital status, once added to conventional risk factors, would improve the cardiovascular risk prediction in CEE/FSU. However, in both MONICA and HAPIEE, the SCORE calibration and discrimination were very similar for the original (non-extended) scale and for the scale extended by education and marital status.

While we did not find relevant external evidence from CEE/FSU, several American and British studies reported no or minimal improvement in the performance of the Framingham coronary risk scale extended by various socioeconomic parameters [59]–[61]. Other studies have shown a better prognostic performance of the cardiovascular risk scales incorporating socioeconomic characteristics (such as ASSIGN and QRISK/QRISK2), compared to traditional instruments (such as the Framingham scale), but they assessed non-nested models [62]–[67] and, therefore, could not directly address the issue of incremental prognostic value of socioeconomic parameters. In this respect, our results are consistent with the negative findings from the studies that used nested models [59]–[61].

### Conclusions

Our main finding – little improvement in the SCORE prognostic performance after inclusion of education and marital status – has several implications. First, our study supported the use of the original, non-extended SCORE in CEE/FSU populations and, therefore, confirmed the important role of conventional risk factors. Controlling them at both the population and individual levels should reduce the CVD burden in CEE/FSU and prevent the reversal of declining CVD rates elsewhere [2], [68], [69]. Second, other sociodemographic and socioeconomic parameters, such as area-level deprivation, when added to SCORE separately or in combination, could independently predict fatal CVD and have incremental prognostic value in specific populations. However, if these parameters are to be routinely used, they need to be easily and objectively measured. Finally, there is a growing interest in the use of extended risk models among individuals at intermediate risk [26], [70]–[72]. The latest ESC guidelines [2], published after our findings were obtained, recommend the novel biomarker measurement and cardiovascular imaging among asymptomatic adults at “moderate risk” (SCORE ≥1% and <5%). Therefore, it is important to investigate whether additional risk factors provide clinically and statistically significant improvement in the SCORE performance among people with intermediate risk levels.
